# Multi-Scale Study of a Phase Change Material on a Tropical Island for Evaluating Its Impact on Human Comfort in the Building Sector

**DOI:** 10.3390/ma17133241

**Published:** 2024-07-02

**Authors:** Lisa Liu, Nadia Hammami, Dimitri Bigot, Bruno Malet-Damour, Jean-Pierre Habas

**Affiliations:** 1 ICGM Institut Charles Gerhardt, University Montpellier, CNRS, ENSCM, 34000 Montpellier, Hérault, France; lisa.liu@umontpellier.fr; 2 PIMENT Laboratory, University of Reunion Island, 97430 Le Tampon, Île de La Réunion, France; nadia.hammami@univ-reunion.fr (N.H.); dimitri.bigot@univ-reunion.fr (D.B.)

**Keywords:** phase change materials, tropical buildings, multi-scale experimentation, thermal comfort, real-world performance

## Abstract

Our study explores the utilization of a phase change material (PCM) to optimize energy efficiency and thermal comfort in buildings in tropical climates. Employing a comprehensive multi-scale approach, this research encompasses both microscopic and macroscopic analyses to rigorously evaluate the PCM’s performance under various environmental conditions. It evaluates the effect of PCMs on ambient conditions in the face of temperature variations and high humidity, utilizing experimental methods at different scales (microscopic and macroscopic). Microscopic analyses reveal the composite structure of the PCM, consisting of microencapsulated paraffin within a cellulose fiber matrix. At a macroscopic scale, experiments using two real-scale test cells evaluated thermal performance and its influence on thermal comfort. Temperature and humidity data were meticulously collected over an extended period to assess the PCM’s impact on indoor regulation. We employed type T thermocouples and flux meters to monitor thermal dynamics and energy flux across the building walls. This setup facilitated a detailed comparison of temperature variations and thermal comfort metrics between the PCM-equipped test cell and a control cell. The results indicate a seasonal duality of the PCM: beneficial in winter for thermal regulation but problematic in summer due to excessive heat retention. The conclusions highlight the importance of carefully selecting and adapting PCMs for tropical climates, thus providing valuable insights for designing sustainable buildings in regions facing similar climatic challenges.

## 1. Introduction

In the current energy and climate context, a significant reduction in greenhouse gas emissions is needed in the coming decades. There is an urgent need to limit the use of fossil fuels and implement energy-saving technological solutions. In France, since 1974, thermal regulations have required standards to be met for construction and renovation in the building industry. One of the standards applied was named RT-2012, which came into force on 1 January 2013 and imposed a primary energy consumption limit of 50 kW h/m^2^/year [1]. It was followed in 2021 by the even more stringent RE-2020 standard [2], modulated according to the climatic conditions of each region.

For instance, on Reunion Island, several additional rules are strongly recommended to optimize the energy consumption of a house, whether for new construction or a renovation project [3]. Indeed, this French Overseas Department has an overall tropical climate. It is very different from what is observed in Metropolitan France. In such conditions, it is expected to find that air conditioning alone accounts for nearly 50% of a household’s electricity consumption. Insofar as 68% of the electricity produced is derived from fossil fuels [4,5], this situation is debatable. First, it negatively impacts the environment due to carbon dioxide emissions, contributing to global warming. Furthermore, the cost of electrical energy, which has been rising steadily for several years, raises significant concerns from an economic point of view. Therefore, enhancing a building’s thermal envelope is a worthwhile, if not necessary, course of action. In this respect, using renewable resources (energy and materials) and increasing energy efficiency are the prominent strategies for achieving these targets. Thermal energy storage (TES), one of many available technologies, may play an essential and complementary role. Indeed, because of its charge and discharge intervals, its operation delays the availability of thermal energy. In other words, recovering and reusing stored energy can cut costs and overall energy consumption under the right circumstances.

## 2. Literature Review

For the last few decades, phase change materials (abbreviated PCM) have been identified as one of the most efficient ways to store thermal energy. This efficiency is due to the potentially large amount of thermal energy supplied or removed from the material to change states (solid–liquid, solid–solid, solid–gas, liquid–gas) [6]. Solid–gas and liquid–gas transitions are very rarely studied in the building domain due to the large volume change during the phase change [7]. Many review articles focus on solid–liquid transition PCMs, as found with the PCM studied in our research, and more recently on solid–solid transition ones. This focus is evident in both review articles and original research studies.

For energy storage purposes, Abhat [8], Farid et al. [9], Nazir et al. [10], Sharma et al. [11], and Yang et al. [12] discuss the potential of solid–liquid PCMs for energy storage across various systems, emphasizing their thermal properties and application in reducing energy consumption. Al-Absi et al. [13] and Lamrani et al. [14] investigate the optimal placement and integration of such PCMs in energy storage systems, focusing on their performance in enhancing energy efficiency. Kenisarin [15], Aftab et al. [16], and Alkan et al. [17] provide comprehensive overviews of solid–solid PCMs for energy storage, detailing their thermophysical properties and potential applications across various energy systems. Du et al. [18] and Aftab et al. [19] examine the properties of solid–solid PCMs for energy storage, focusing on their heat storage capacity and thermal stability under different conditions.

For building energy storage, Pielichowska and Pielichowski [7], Zalba et al. [20], Khudair et al. [21], Zhang et al. [22], Zhou et al. [23], Song et al. [24], da Cunha et al. [25], and Cabeza et al. [26] highlight the efficacy of solid–liquid PCMs in enhancing thermal comfort and energy efficiency in buildings by stabilizing indoor temperatures. Additionally, Jeon et al. [27] and Ben Romdhane et al. [28] examine the use of such PCMs in reducing energy consumption in buildings, demonstrating their effectiveness in passive cooling and thermal energy storage. Ahmadi et al. [29] and Cao et al. [30] demonstrate their effectiveness in improving thermal energy storage and reducing energy consumption using solid–solid PCMs.

Integrating PCMs into building components or materials is necessary to take advantage of their benefits. Kuznik et al. [31] and Soares et al. [32] review the integration of PCMs into building materials, detailing their impact on energy efficiency and the thermal regulation of indoor environments. Akeiber et al. [33] and Faraj et al. [34] experimentally evaluate the application of PCMs in construction materials, highlighting their benefits in energy savings and improving thermal comfort. Liu et al. [35] and Du et al. [36] review the incorporation of solid–solid PCMs into construction materials, highlighting their benefits for enhancing the thermal efficiency of buildings. Lee et al. [37] studied the integration of a thin phase change material on a building wall to reduce heat transfer through it. They showed the potential of this solution to reduce building energy needs by regulating inside air temperature and heat transfer through the wall. They also validate their model and suggest that the location and thickness of the PCM layer can be optimized.

To use these materials effectively, it is critical to precisely understand their behavior, to characterize them, and to be able to design them. Kenisarin [15], Aftab et al. [19], Murrill and Breed [38], Zhang et al. [39,40], Xi et al. [41], Busico et al. [42], Li et al. [43], Gao et al. [44], and Barreneche et al. [45] provide detailed experimental data on the characterization of PCM, analyzing their thermal properties and phase transitions. Several other papers present ways to design a phase change material, such as Cao et al. [30], Aftab et al. [19], Liu et al. [35], Du et al. [36], Xi et al. [41], and Lu et al. [46].

The latter does not have leakage issues compared to solid–liquid PCM. However, for the relevant application temperatures in the building industry, the associated latent heat is insufficient for optimal PCM use. Thus, solid–liquid phase change is the most suitable process for integrating the building’s envelope.

Among solid–liquid PCM, Cabeza et al. [26] and Barreneche et al. [45] identified applications according to the phase change temperature range:

1. PCMs with melting temperatures lower than 21 °C can be used for cooling functions in buildings;
2. PCMs with melting temperatures comprised between 20 and 30 °C are suitable for indoor thermal comfort in buildings;
3. PCMs having a melting temperature between 29 and 60 °C can be used in energy storage, in particular for hot water production;
4. Finally, PCMs with a melting temperature higher than 60 °C are dedicated to the heating function.

Incorporated into polymer-based building materials [46,47,48,49,50,51,52,53,54,55,56], gypsum or plaster [51,57,58,59,60,61,62,63,64], concrete [65,66,67,68,69,70], or metal [66,71,72,73,74,75,76,77,78,79,80,81,82], PCMs enable thermal energy from solar activity to be captured directly and diffused later when thermal conditions have changed. They also contribute to human comfort by reducing fluctuations in indoor air temperature and keeping the temperature closer to a desired level. PCMs fit perfectly with a variety of current insulation solutions. As a result, they are an appropriate answer to tightening thermal standards in modern or refurbished buildings, which are frequently built with lightweight frames for economic reasons.

Our current project, MCP-iBAT, seeks to define and optimize biobased phase change materials for use in building applications on Reunion Island. These PCMs must be sized considering the environmental and climatic constraints specific to the tropical climate zone, where temperatures and relative humidity can be quite significant. Hence, this paper is not solely concerned with determining the thermal performance of a commercial product on a laboratory scale or in a pilot plant. It compares macroscopic data to the characteristics of the material previously determined by various techniques, ranging from chemical structure and composition to physicochemical properties. Furthermore, unlike many other studies, this one focuses on the PCM’s influence on the room’s interior air temperature rather than the walls’ surface temperature. This approach is much more consistent and realistic with the actual conditions of future material use. Indeed, the objective is to improve the user’s thermal comfort based on interior air quality. Ideally, a study should establish precise relationships between the micro-properties of a material and its macro-performance under conditions close to those encountered in the desired application to effectively evaluate its real interest and understand the mechanisms involved in its action.

## 3. Materials and Methods

### 3.1. Material

The PCM used in our experiment is commercialized by MCI Technologies (Technopôle Saint-Brieuc Armor 5, rue Sophie Germain, 22440 Ploufragan, France) under the trademark Enerciel^®^. To rigorously analyze the results, we characterized its thermal and chemical properties to assess the reliability of the information in the product datasheet. Then, on-site thermal data were collected to evaluate the performance of the materials under conditions that would simulate their actual use in a macroscopic environment.

### 3.2. Scanning Electron Microscopy

The commercial PCM was structurally characterized using a scanning electron microscope SEM from Hitachi 2600N (Tokyo 105-6409, Japan) working with secondary electrons at 20 kV. Before analysis, the surface of the PCM sample was metalized with gold (5 nm thick) using the sputtering technique.

### 3.3. Chemical Analyses

The chemical structures of the composite PCM and its constituting elements were analyzed using a Fourier Transform Infra-Red spectrometer (FTIR). The Spectrum One apparatus from Perkin Elmer (Waltham, MA, USA) was equipped with a Universal Attenuated Total Reflectance accessory made with a ZnSe crystal. This equipment enabled the chemical analysis of the various samples, in solid or liquid form, by direct deposit upon the crystal surface without any prior dissolution, dilution, or mechanical grinding. When studying solid samples, a lever arm presses the material to be analyzed against the crystal to optimize the physical contact between both substrates and the signal quality recorded. The spectra were registered by varying the wavenumber between 4000 and 650 cm^−1^ using 32 scans with a 2 cm^−1^ resolution.

### 3.4. DSC Experiments

The PCM’s calorimetric properties were evaluated as a function of temperature using StarOne^®^ equipment from Mettler Toledo (Columbus, OH, USA). This Differential Scanning Calorimeter (DSC) was first calibrated for heat flow and temperature using indium as a reference (melting temperature = 156.6 °C and latent heat = 28.47 kJ kg^−1^). The PCM sample (ca. 10 mg) was placed in an aluminum pan with a perforated lid to avoid any risk of leakage provoked by the over-pressure effect during the analysis. An empty crucible was used as an inert reference. After filling, the capsule was sealed and placed in the apparatus’s instrumented oven at room temperature.

The subsequent experimental protocol, consisting of four consecutive steps, was carefully followed:

(i) The sample was initially cooled from ambient temperature to −70 °C using a −5 °C min^−1^ thermal ramp;
(ii) It was maintained at −70 °C for 15 min to ensure its thermal equilibrium;
(iii) Then, the thermal profile was recorded as a function of the temperature, with a constant heating ramp *β*, up to 100 °C in an inert atmosphere. This analysis section enabled us to determine the PCM’s melting characteristics (onset and endset temperatures and latent heat);
(iv) Ultimately, the PCM sample was cooled down to −70 °C using a thermal ramp of −*β* (i.e., opposite to the value used during the heating step). In this way, the crystallization properties of the material were measured accurately.

These analyses were performed with fixed *β* values of 0.3, 1, 3, and 5 °C min^−1^.

### 3.5. Thermogravimetric Analyses

The thermal stability of the commercial PCM was evaluated using a Mettler Toledo TGA2^®^ thermogravimetric analyzer (Columbus, OH, USA). This instrument was retained as it is suitable for relatively large sample sizes, which is important for characterizing multi-component systems. The specimen (ca. 4 g) was set directly in an alumina support plate under an airflow rate of 50 mL min^−1^. The heating ramp was set at 10 °C min^−1^ from 25 °C to 900 °C.

### 3.6. Thermomechanical Experiments

The viscoelastic properties of the PCM were registered using a stress-controlled dynamic rheometer (MCR 301^®^ from Anton Paar, Graz, Austria). The experiment, currently named the thermomechanical test, consisted of registering the evolution of the complex shear modulus G* = G′ + j G″ as a function of temperature with a fixed angular frequency (*ω* = 1 rad s^−1^) and a heating ramp of 3 °C min^−1^. The actual component G′, called the “storage modulus”, is specific to the elastic contribution of the sample. In other words, it is proportional to its mechanical rigidity. The imaginary part G″, classically named the “loss modulus”, refers to the dissipated mechanical energy. The characterization of the sample in the solid state using rectangular torsion geometry under constant strain (0.8%) was chosen after the definition of the linear domain. To this end, a parallelepiped-shaped sample was cut from the coating layer with dimensions of 35 mm × 10 mm × 1.5 mm.

### 3.7. Outdoor Environment Description

**Figure 1:**
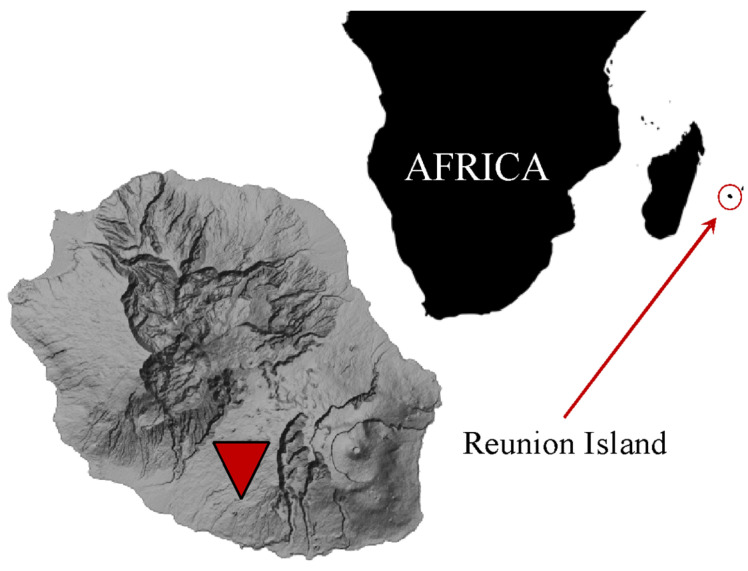
Location of Reunion Island and the experimentation site (red symbol).

Located in the southern hemisphere (Figure 1), near the Tropic of Capricorn, Reunion Island benefits from sunshine with positive radiative forcing, which tends to warm the environment (i.e., more energy received than emitted) [83]. It is equivalent to that of southern Algeria, Mexico, or Vietnam. The ambient air in Reunion Island is qualified as humid because the humidity rate in winter for coastal areas is around 50–55% against 60–70% for the highs (sites at higher altitudes). In summer, this rate reaches 80 to 95%. The percentage increases with altitude due to temperatures approaching the dew point. The “hygrometry-temperature” relationship explains why fog exclusively affects the highlands. The Humidex index, which represents comfort and discomfort in a given setting, is defined by relative humidity and temperature. When combined with the wind, this model forms the concept of the felt temperature, which varies constantly from one area of the island to another [83].

A given place’s daily and annual thermal amplitude rarely exceeds 10 °C. The maxima and minima are recorded, respectively, during the austral summer and winter. The lowest temperature is measurable between 6 and 7 a.m. and corresponds to the maximum cooling point of the night.

Conversely, around 1 p.m., the sun warms the ground and air as much as possible. The highest temperature was recorded at Le Port on 6 March 2005 (36.9 °C). The Bellecombe station (2245 m) recorded the lowest, around −5 °C, in 1975 [83].

**Figure 2:**
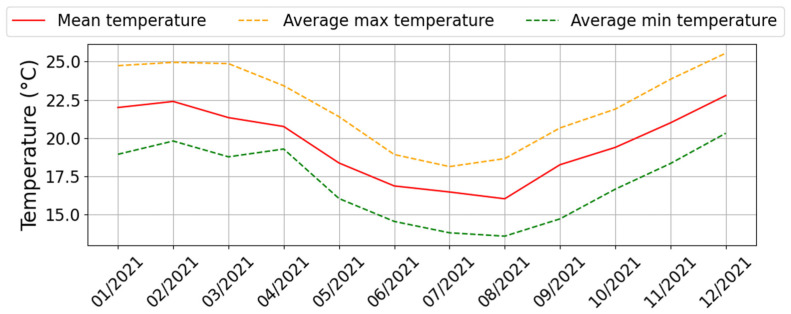
Annual outdoor air temperatures in Le Tampon, Reunion Island (France).

The experiment is located in Le Tampon (−21.27, 55.5), south of the island. According to the criteria of Köppen–Geiger’s climate classification [84], Le Tampon belongs to the Cfa climate zone, a temperate climate without a dry season and with a hot summer. Alternatively, it is also defined as a humid subtropical climate. Figure 2 shows the average monthly temperatures of outdoor air registered in 2021. The mean temperature fluctuated between 16 °C and 23 °C, with the coldest and hottest months being February and August, respectively.

### 3.8. Pilot Station

**Figure 3:**
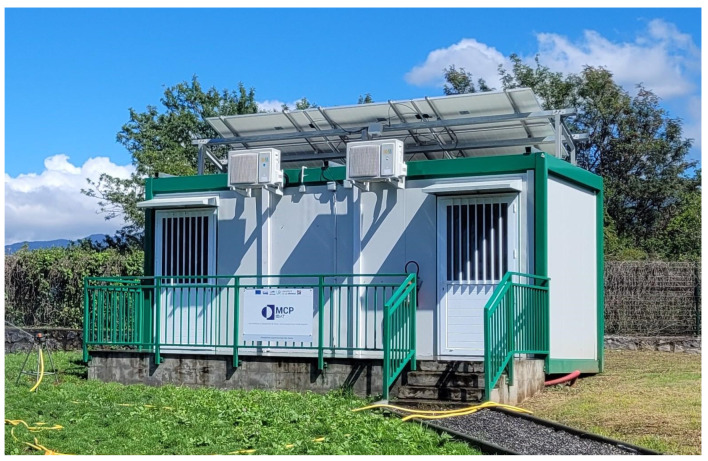
The platform in Le Tampon, Reunion Island (France).

**Figure 4:**
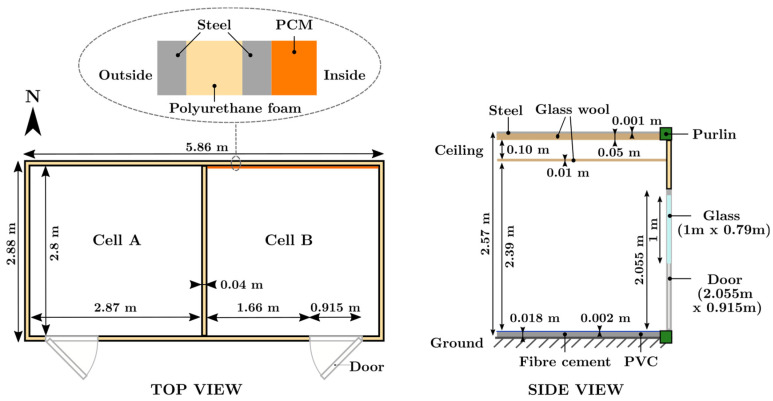
Experimental setup.

The experimental platform in Figure 3 has the following dimensions: 2.88 m × 5.96 m × 2.57 m. It comprises cells A and B, as shown in Figure 4. Cell A is the reference compartment, while cell B is the test room. The load-bearing walls comprise a layer of polyurethane between two layers of galvanized steel. Table 1 summarizes the materials’ physical characteristics from the manufacturer’s technical documentation.

The commercial PCM (named Enerciel and defined above) was used to equip cell B, particularly the surface of the north wall (6.86 m^2^). With the initial appearance of a paste, it was applied with a notched spatula to adjust the coating’s thickness between 3 and 5 mm. The north wall was chosen because it is more exposed to solar radiation throughout the day (southern hemisphere).

### 3.9. Data Collection

The data collection began in August 2020 and is still ongoing. Type T thermocouples, with ±0.1 °C accuracy, are used to measure the temperature of:

- The indoor air;
- The inner surface of the walls, ceiling, and floor;
- The outer surface of the north wall.

Furthermore, operative temperature and relative humidity are measured with a Testo 175H1 positioned in the middle of the cell. We also affixed HFP01SC flux meters from Hukseflux to the interior surface of all vertical walls and the ceiling to measure conductive heat flux. These multiple sensors are connected to a CR3000 data logger, sampled at 0.1 Hz, and averaged per minute.

### 3.10. Thermal Performance Analysis

This paper presents three cases: a summer day case, a winter day case, and a mid-season day case. As environmental parameters vary according to weather conditions, we selected the three clearest days based on the ratio of diffuse to global daily irradiance. Therefore, the selected days are summarized in Table 2.

To analyze the PCM’s performance and its suitability in the experimental structure, we computed two indicators:

- The efficiency of the exchange of thermal energy through the north wall with the PCM;
- The intensity of thermal discomfort (ITD).

This latter characteristic has been defined in previous work by John Evola et al. [85] for evaluating the performance of a PCM. Under its initial form, the intensity of thermal discomfort (ITD) was the integral of the positive difference between the current temperature and the upper threshold for comfort throughout P. But Castell and Farid [59] later proposed integrating the difference over the whole day by considering the lowest threshold of the comfort zone.

The comfort zones are established by two major standards: ASHRAE Standard 55 (the American national standard) [86] and EN 15251 (the European standard) [87]. As Reunion Island is a French Overseas Department and Region, the European standard has been retained in our study, with the following assumptions:

- Air velocity = 0.2 m s^−1^;
- Clothing = 1.3 clo (trousers, shirt, sweater, and jacket);
- Metabolic rate = 1 met (seated with sedentary activity);
- Average radiant temperature = 23 °C;
- Relative humidity = 50%.

The thermal comfort zone is set between 23 and 26 °C, with a predicted mean vote (PMV) of ±0.2. This latter characteristic, developed by Fanger [88], represents the mean thermal sensation vote on a standard scale for a large group of people for any given combination of thermal environmental variables, activity, and clothing levels [89]. In this scale, the 0 value corresponds to the neutral sensation.

## 4. Results and Discussion

### 4.1. Structural and Chemical Analyses

#### 4.1.1. Scanning Electron Microscopy (SEM)

**Figure 5:**
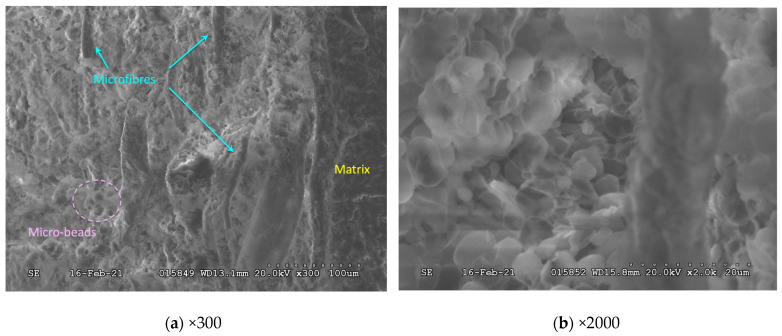
SEM analysis performed on the commercial PCM.

The first SEM images recorded on the PCM were taken at low magnification (×120). Under these conditions, the PCM presented a structure like a composite material. Indeed, it appeared to combine a matrix and short fibers (length > 250 μm). The latter were randomly distributed, with irregular diameters reminiscent of natural fibers. At higher magnification (×300, Figure 5a), it was possible to detect micro-beads in addition to the matrix and fibers mentioned above. The fibers and micro-beads were significantly more noticeable when magnified to ×800. However, it was difficult to determine whether the micro-beads were homogeneous and solid or reservoir-type micro-spheres. At the highest magnification (×2000, Figure 5b), it could be stated with certainty that the spheres were hollow and not made of a single material. Some were even found to be punctured. This result suggested that the PCM studied was most likely micro-encapsulated, the micro-beads resulting from an emulsion polymerization under mechanical agitation. Note that their diameter was evaluated between 4 and 10 μm.

#### 4.1.2. Fourier Transform Infrared Spectroscopy

**Figure 6:**
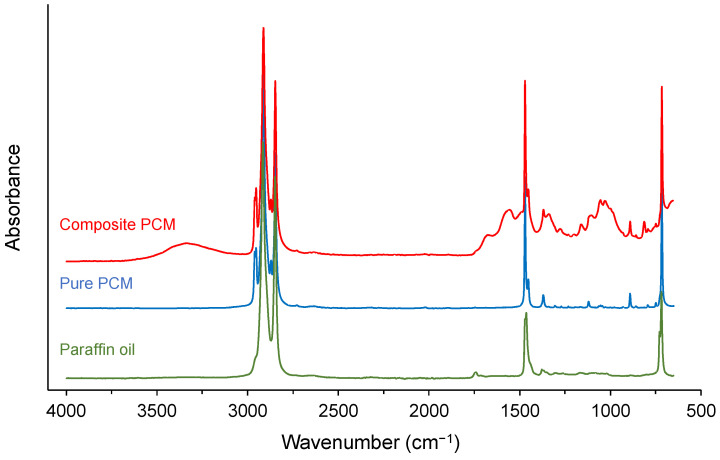
FTIR spectra of commercial composite PCM, remaining liquid after the first analysis, and paraffin oil.

The chemical structure of the commercial PCM was studied using FTIR spectroscopy. The resulting spectrum, presented in Figure 6, comprises multiple absorbance bands, which combine the specific responses of the different constituting elements of the PCM. At first glance, the direct interpretation of the spectrum may seem a little difficult.

However, it is essential to point out that at the end of the experiment, an oily deposit was observed on the surface of the FTIR spectrometer crystal, despite the initial sample being perfectly dry. The characteristic spectrum of this single liquid was then registered and is presented in Figure 6. It is clear that the absorbance bands that make up this spectrum are also present in the spectrum of the commercial PCM. As a result, the leakage of this oily liquid was likely caused by the mechanical pressure of the arm used to hold the sample in place. The most plausible hypothesis is that this liquid was initially contained in the micro-spheres as described above. This is most likely the “pure PCM,” while the commercial PCM should be referred to as “composite PCM” to avoid confusion. Assigning the absorbance bands contained in the spectrum of a pure PCM was quickly carried out using spectral data tables. They are characteristic of the vibration of only aliphatic C-H bonds in three modes: stretching (2800–3000 cm^−1^), bending (1380–1480 cm^−1^), and rocking (680–760 cm^−1^). Such a hydrocarbon structure is characteristic of molecules belonging to the alkane category, specifically paraffin, as shown in Figure 6. These compounds come from petrochemicals. In other words, the “pure PCM” is not derived from biomass. To support this assertion, it is essential to point out that the closest biobased chemical structures are fatty acids and esters. However, the latter contains a carbonyl function that produces a very intense absorbance peak in the 1600–1800 cm^−1^ region, but nothing like this is detected in the spectrum of pure PCM. On the other hand, further identification of the composite PCM components revealed that the fibers in the binder were of plant origin. Indeed, the FTIR spectral response of cellulose appears in the overall spectrum. This characteristic undoubtedly leads the producer of the commercial PCM to declare that their product is biobased.

### 4.2. Evolution of Thermal and Thermomechanical Properties with Temperature

#### 4.2.1. Differential Scanning Calorimetry (DSC)

The thermal characteristics of the commercial PCM were evaluated using Differential Scanning Calorimetry (DSC). For this purpose, three samples were collected from the experimental installation and analyzed according to the procedure described in experimental Section 3.4. The data recorded showed high repeatability. The reported melting and crystallization temperatures and associated enthalpies are averaged over the three tests (Table 3).

**Figure 7:**
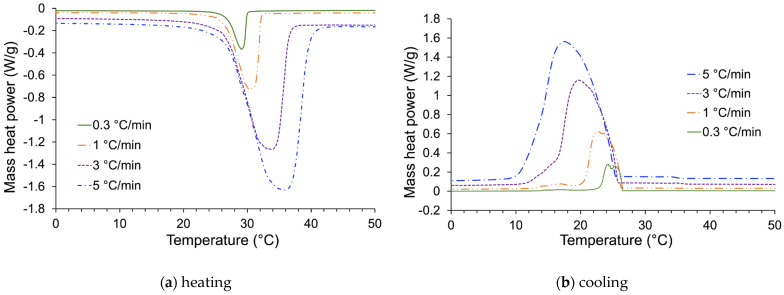
DSC analyses of the commercial PCM with different heating and cooling rates (5, 3, 1, and 0.3 °C min^−1^).

Figure 7a presents the results registered in heating mode for different thermal ramps *β* comprised between 0.3 °C min^−1^ and 5 °C min^−1^. This figure indicates that the onset of the PCM melting zone is independent of the heating rate (about 27 °C). This is consistent with the fact that fusion is a non-kinetic process. Inversely, the end of the melting zone is influenced by the thermal ramp used during the DSC experiment. However, integrating the melting peak for each heating ramp yields an enthalpy value that is constant overall (i.e., comprised between 160 and 170 kJ kg^−1^).

The crystallization process is described using cooling mode analyses that show the presence of an exothermic peak (Figure 7b). With an increase in the heating ramp, the onset of this phenomenon shifts to a lower temperature zone. This evolution is inherent to the kinetic character of crystallization [90,91,92]. In other words, the spatial organization of the molecules constituting the PCM does not occur instantaneously but at a very specific rate. However, this process seems complete for all studied heating rates. The value is of the same absolute order of magnitude as that recorded with melting, attesting to the very good reversibility of these phenomena. Nevertheless, these registered enthalpic values were slightly lower than the commercial data (184 kJ kg^−1^).

#### 4.2.2. Dynamic Rheometry

**Figure 8:**
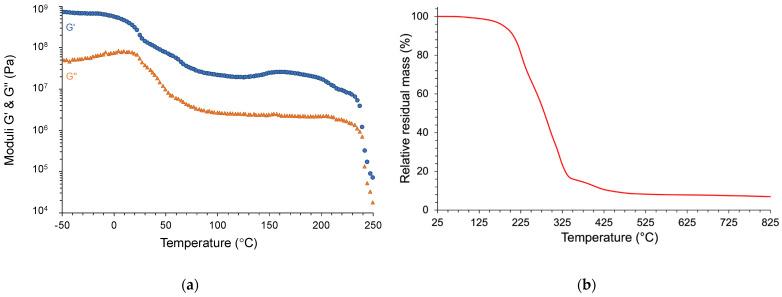
Thermomechanical analysis of the commercial PCM (**a**) and its thermogravimetric analysis (**b**).

The viscoelastic properties of PCM, initially in the form of a small plate of regular dimensions, were evaluated using rectangular torsion geometry. The corresponding thermogram is shown in Figure 8a. In the low-temperature region (T < 25 °C), the material shows a high mechanical rigidity (G′ > 8.10^8^ Pa). Then, both moduli curves show a deflection for T > 25 °C. This temperature value coincides with the melting onset of the “pure” PCM, as seen previously with the DSC analyses. However, the “composite” PCM does not flow at the macroscopic level. It simply softens, with a predominance of elastic behavior (G′ > G″). Indeed, the “pure” PCM, which becomes liquid, remains trapped in micro-beads surrounded by a matrix reinforced with cellulose microfibers. Following this transition, the rheological properties change slightly to a temperature of 230 °C. Above this temperature, both graphs exhibit a sharp drop, which is typical of viscoelastic moduli values. There are two plausible origins for this evolution:

1. Melting or softening of the binder (matrix in which the micro-spheres are dispersed);
2. Thermal degradation of the composite material.

The thermogravimetric analysis (TGA) of the composite PCM was used to determine which hypothesis needs to be validated.

#### 4.2.3. Thermogravimetric Analysis (TGA)

The characterization of the commercial coating by thermogravimetric analysis performed in dynamic mode enabled us to clearly define that the drop in moduli recorded above 230 °C was not representative of any matrix softening (Figure 8b). Indeed, in this same temperature range, the sample mass decreased drastically, thus revealing the beginning of its thermal degradation. In other words, hypothesis 2 must be retained as valid.

At higher temperatures (up to 825 °C), the residual mass was not equal to zero. Under these severe thermo-oxidative conditions, the organic substances (PCM, micro-beads, and cellulose fibers) are eliminated. Then, the observed residue reveals the presence of a mineral or metallic material (up to 6% by mass).

In conclusion, the analysis using different experimental techniques showed that the Enerciel coating was not a material based on a single compound. Instead, it should be described as a composite PCM with several immiscible components. The active compound (“pure” PCM) is a petroleum-derived paraffin contained in micro-beads for thermal applications. The surrounding matrix is partially made up of cellulose fibers and a thermally stable inorganic compound.

### 4.3. Onsite Data Analysis

**Figure 9:**
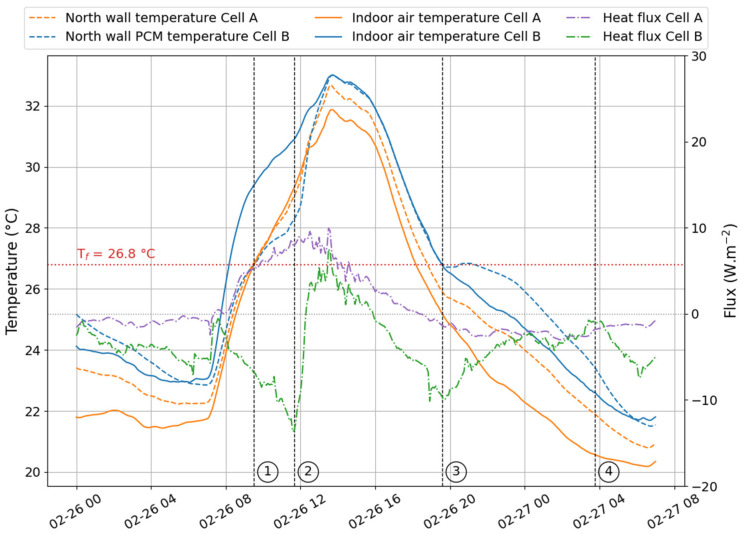
Heat flux and temperature observed on the north wall, in Le Tampon (26 February 2021).

**Figure 10:**
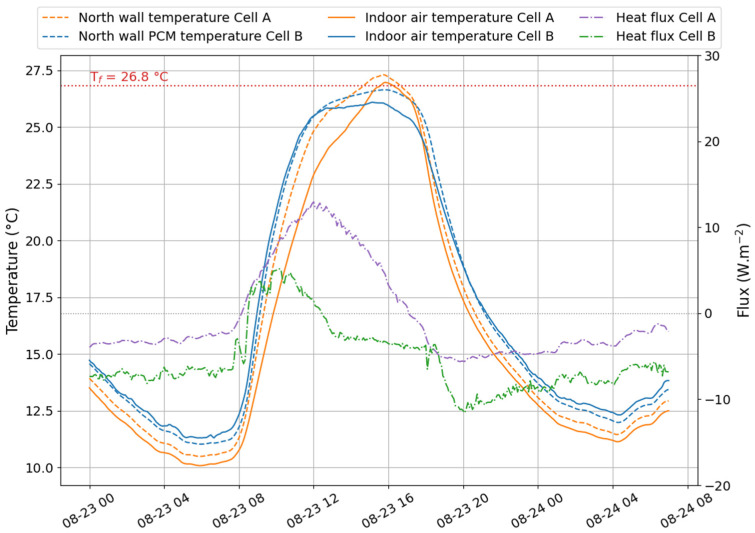
Heat flux and temperature observed on the north wall, in Le Tampon (23 August 2021).

**Figure 11:**
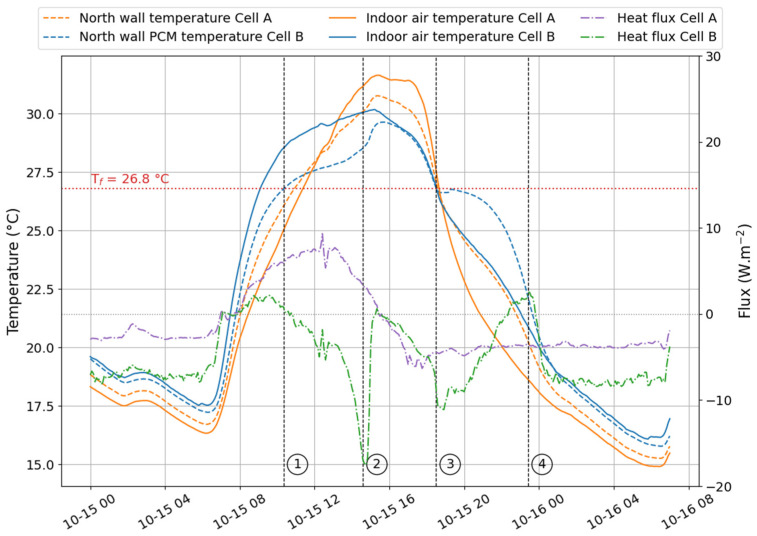
Heat flux and temperature observed on the north wall, in Le Tampon (15 October 2021).

In Figure 9, Figure 10 and Figure 11, we represented the north wall surface temperature in cell A (reference) and cell B (test) and the indoor air temperatures of both cells. In addition, the total heat flux was plotted to illustrate the energy exchange between the outside and inside environments. When the values are negative, the heat flows from inside to outside the wall. Conversely, when the heat flux values are positive, the outdoor environment provides more heat to the indoor environment. The numbers ① to ④ delineate the phase change zones that will be determined in the following paragraphs.

#### 4.3.1. Temperature

Through the DSC analysis, we determined the mean value for the onset of the melting was about T_m,onset_ = 26.8 °C, while the value characteristic of the onset of crystallization was T_c,onset_ = 26.4 °C. These temperatures were used in Figure 9, Figure 10 and Figure 11 to indicate the limits of each phase change zone. It is given by the intersection of T_m,onset_ and T_c,onset_ with the surface temperature of the north wall in cell B (i.e., equipped with the PCM). ① is then the melting onset, while ③ corresponds to the beginning of freezing. It is important to notice that the crystallization onset on 26 February 2021, and on 15 October 2021, observed on the graphs, were respectively 26.7 °C and 26.6 °C. During 30 min, the temperature was kept constant before increasing to 26.8 °C. This phenomenon may be attributed to the supercooling effect.

**Figure 12:**
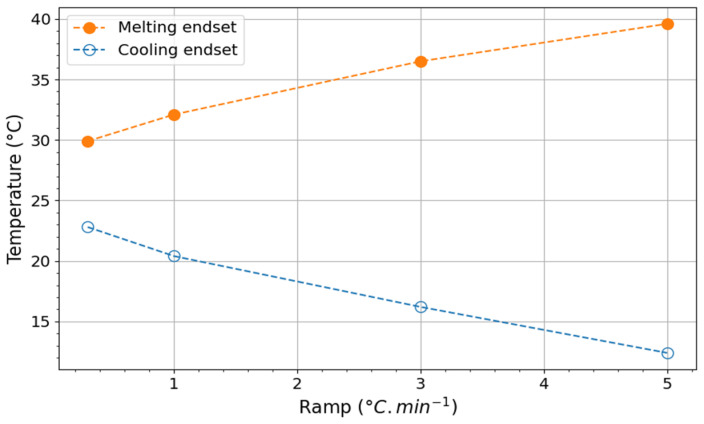
Influence of the thermal ramp on the temperatures associated, respectively, with the endset of melting and crystallization as measured by DSC.

Two techniques can be used to determine the end of a phase change. First, the curves plotted in Figure 12 allow the extrapolation of the temperatures for a temperature ramp *β* close to that of the working environment (i.e., close to 0.01 °C min^−1^). In these latter conditions, the melting endset is 29.1 °C, and the crystallization endset is 23.4 °C. These values approach the temperature at which the phase change begins. In other words, the phase changes will produce themselves over a very narrow temperature range. The second method is based on the heat flux graph of cell B equipped with the PCM. Indeed, similarly to the DSC, when the PCM melts, it absorbs more heat than the other materials in the same environment. It is observable in Figure 9 and Figure 11, where the heat flux decreased above −10 W.m^−2^ for mid-season and summer days, respectively, on 26 February 2021, and 15 October 2021.

Over these two days, we observed the final phase of the fusion process within cell B (the test cell). This turning point was characterized by a stabilization of heat flux, which ceased to decrease and adopted a profile like that observed in cell A (the reference cell). This specific phase was identified as the conclusion of the PCM’s fusion, denoted as ② on the figures.

This result indicates that the phase-change material had reached its maximum capacity for absorbing thermal heat. From this point on, it functioned as a standard material, storing heat according to its thermal capacity while efficiently conducting it through its thermal conductivity.

A similar observation was made during crystallization, marked as ④, when the heat flux increased to a nominal value before the onset of a new cycle (indicated by a decrease in flux). This phenomenon marked the end of the PCM’s thermal discharge.

These graphical analyses allow us to identify the final phase-change temperatures recorded on 26 February 2021, and 15 October 2021. These values are documented in Table 4.

Note that on 23 August 2021 (see Figure 10), the PCM did not fully melt because the environmental conditions did not allow the onset of the melting temperature to be reached.

Based on our graphical visualization results and as summarized in Table 5, the temperatures marking the conclusion of the melting process are lower than those predicted by the regression curve. This discrepancy highlights the margin of error associated with the estimation. Conversely, the temperatures corresponding to the conclusion of the solidification process exhibit better alignment between the two methods, primarily due to the measurement error of the entire acquisition system being approximately ±1 °C.

#### 4.3.2. Heat Flux

**Figure 13:**
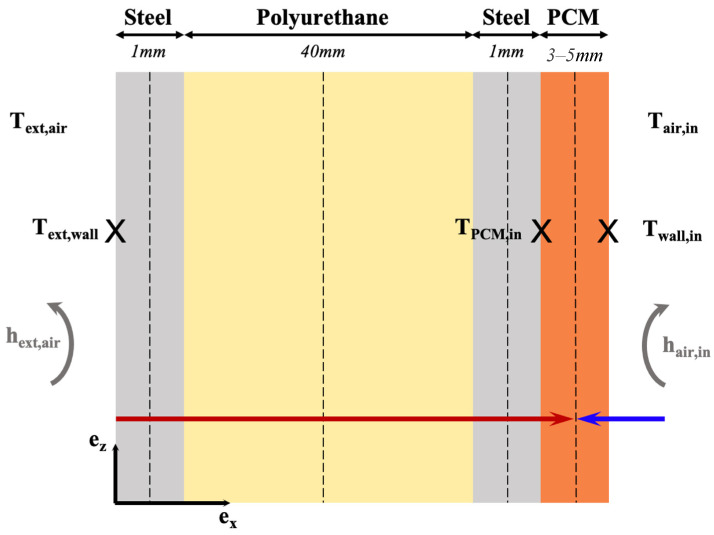
Representation of the thermal fluxes through the north wall equipped with the PCM.

The diagram in Figure 13 schematically represents the directions of the thermal fluxes across the north wall with a PCM coating during the heating phase. The left side of the wall is exposed to outdoor conditions, where T_ext,air_ and T_ext,wall_ are well-established parameters. On the opposite side, the PCM was applied as a coating to the wall’s surface. Thermocouples were strategically positioned on either side of the coating, and indoor air temperature was recorded. The red arrow symbolizes the heat flux originating from the external environment, while the blue arrow represents the heat flux emanating from the indoor surroundings.

Hence, we posited that the predominant source of thermal energy exchange within the PCM stemmed from the heat generated by the indoor air.

In the morning, the east wall receives more heat than the other walls, owing to the sunrise. Consequently, cells A and B exhibit slightly divergent behaviors, with cell A being more exposed to the sun’s rays at day’s end through the west and north walls. This phenomenon elucidates the disparity in indoor air temperatures between cell B and cell A during the morning hours.

### 4.4. Analysis of Environmental Variables and Thermal Comfort

#### 4.4.1. Selection of Study Periods

**Figure 14:**
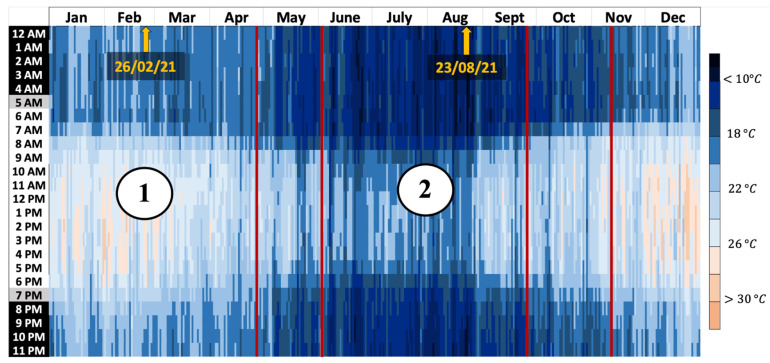
Annual evolution of the outdoor air temperature and illustration of the studied periods.

The investigation into thermal comfort will rely on climatic periods typical of the upper island region, as depicted in Figure 14:

- The summer season (January to April) noted “1”.
- The winter season (June to September) noted “2”.
- Two seasonal clear-sky days with maximum solar irradiance:
  - a summer day (26 February 2021);
  - a winter day (23 August 2021).

The selection of the study days was based on their representation of the most challenging conditions for both the summer and winter seasons, providing a robust basis for assessing the effectiveness of phase change materials (PCMs) in diverse climatic scenarios. Specifically:

- For summer, we chose the hottest period of the summer season, focusing on a day characterized by clear skies, high solar radiation, and low wind speeds. These conditions were selected because they represent extreme summer scenarios that impose significant thermal loads on building envelopes, challenging the PCM’s capacity to mitigate heat gain.
- In contrast, the winter day selected had overcast skies and higher wind speeds, typical of winter conditions where heat loss is more pronounced. This day was chosen to reflect a scenario where the PCM’s ability to reduce heat loss is crucial. Additionally, this day exhibited higher heat fluxes through the exterior walls, which were critical for evaluating the PCM’s performance in minimizing heat loss during colder periods.

By selecting days with these specific characteristics, we ensured that our study encompassed a range of extreme conditions that are critical for understanding the performance of PCMs in improving thermal comfort and energy efficiency in buildings.

#### 4.4.2. Method of Analysis

In our initial approach, we will examine physical parameters individually. We will assess the PCM’s capacity to influence operating temperature and relative humidity for each specific period. This segmented analysis of these parameters enables us to pinpoint the PCM’s impact on a single sensory aspect, such as a temperature-modulated air conditioning system.

Subsequently, we will calculate the potential utilization duration of active heating (during winter) or cooling (in summer) devices, both with and without the PCM, to ascertain potential energy savings. For this purpose, we will adhere to the recommendations provided by the Energy Code [93] concerning set temperatures: 26 °C for cooling and 19 °C for heating.

However, this specific approach may not provide a comprehensive assessment of the combined effects of these variables on a multi-sensory system like the human body. Therefore, we aim to evaluate the influence of the PCM on thermal comfort by conducting a comprehensive analysis of three critical environmental parameters: operating temperature (incorporating air temperature and average radiant temperature), relative humidity, and air velocity.

In a confined environment, thermal comfort can be defined as a state of thermal neutrality in which individuals do not express a need for cooling or heating [94]. In such conditions, thermoregulatory mechanisms remain inactive. Nevertheless, there exists a zone where variations in thermal comfort are minimal. This zone is commonly referred to as the “thermal comfort range” and has served as a foundational concept for numerous studies in the field.

In humid tropical climates, Givoni’s study [95] is a foundational reference frequently consulted by building engineers. It introduces a graphical approach to understanding an individual’s thermal sensation. The “summer” diagram is rooted in the physiological concept of evapotranspiration and is particularly relevant for sedentary activities and light clothing, which are common on Reunion Island during this season. This diagram establishes correlations between operating temperature and relative air humidity, defining comfort ranges that can be expanded with air movement. Three distinct comfort zones emerge, each associated with varying air velocities ranging from 0 m s^−1^ to 1 m s^−1^. Beyond 1.5 m s^−1^, air velocities are considered excessive and may result in drafts. Consequently, this approach advocates passive building design without needing HVAC systems.

The “winter” diagram similarly delineates three comfort zones, integrating passive heating solutions, with relative humidity consistently maintained between 20% and 80%. Temperature variations span from 19 °C to 26 °C without additional strategies. This thermal comfort range can be extended by adjusting temperatures, which can themselves be influenced by the introduction of internal heat sources (ranging from 11.5 °C to 26 °C) or passive solar heating (from 7 °C to 26 °C). The approach is designed for sedentary activities and assumes average clothing suitable for winter conditions.

This methodology was developed based on measurements collected using the autonomous thermo-hygrometers described in Section 3.9.

#### 4.4.3. Summer Day: 26 February 2021

We are investigating the impact of a PCM on temperatures, humidity, and the occurrence of thermal comfort on a summer day (26 February 2021).

**Figure 15:**
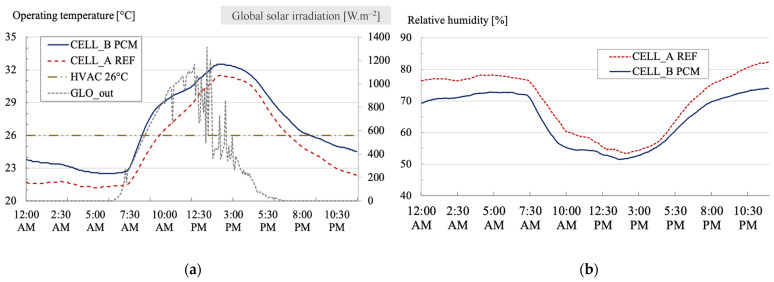
Evolution of operating temperature (**a**) and relative humidity (**b**) inside the test benches during the summer day (cell A: without the PCM; cell B: with the PCM).

Figure 15 enables us to trace the trajectory of operating temperatures within the two test cells. The operating air temperatures are generally higher in the cell with the PCM than in the reference cell (without the PCM). On average, they reached 26.8 °C for cell B (with the PCM) instead of 25.2 °C without the PCM. The maximum temperatures recorded in the cell without the PCM were 31.5 °C, while they reached 32.5 °C with the PCM.

The difference between the cells is also negligible when examining the minimum temperatures reached: 21.2 °C without the PCM versus 22.5 °C with the PCM. The most notable temperature difference between the two cells was 2.8 °C, favoring the cell without the PCM.

This parameter helps us understand that the temperatures remain relatively cool during the summer.

The representation of global solar irradiation provides insights into the primary source of heating for the cells, which is solely attributed to solar radiation. This observation indicates the absence of additional heat contributions from the immediate environment. In the afternoon, the sky becomes overcast, as evidenced by the significant reduction in solar irradiation (Figure 15). This pattern is characteristic of the summer season and is closely tied to the island’s wind patterns, driven by its rugged and steep terrain. These conditions give rise to thermal breezes that carry clouds from the ocean to higher elevations.

The relative humidity within the cell equipped with the PCM is lower than in the reference cell. On average, it was 64.6% for cell B, as opposed to 69.5% without the PCM. The maximum relative humidity recorded in cell A was 82.3%, whereas it reached 74.0% with the PCM. Regarding minimum relative humidity levels, cell A registered 53.3%, compared to 51.5% with the PCM.

Given that both cells are airtight, we can deduce that the nature of the PCM matrix (cellulose) absorbs moisture from the air. This moisture absorption is particularly evident at the beginning and end of the day, with a maximum difference of nearly 9% in favor of cell B.

This behavior is characteristic of porous materials with hygroscopic properties, such as earth, plaster, or concrete.

Figure 15 illustrates an active cooling system’s summer set point temperature. The theoretical set point, which is typically at 26 °C, aligns with the phase change temperature of the PCM in our case. Given that temperatures are generally higher in the cell with the PCM, the potential operational duration of an air conditioning solution would also increase to 12 h for cell B compared to 9 h for the reference cell.

This observation raises important considerations regarding the suitability of using such a PCM across all climatic zones of the island. It highlights the importance of selecting PCMs with properties tailored to the specific environmental conditions and intended use during the design phase [96].

It is important to note that the phase change of the coating, which occurs at 26 °C, has a negligible impact on the operating air temperature. The thickness of the coating (<1 cm) and its surface distribution (16% of the apparent interior surface) are too small to significantly influence the air volume within the cell (23 m^3^). However, the “hygroscopic buffer” effect is noteworthy, particularly in regulating indoor relative humidity.

**Figure 16:**
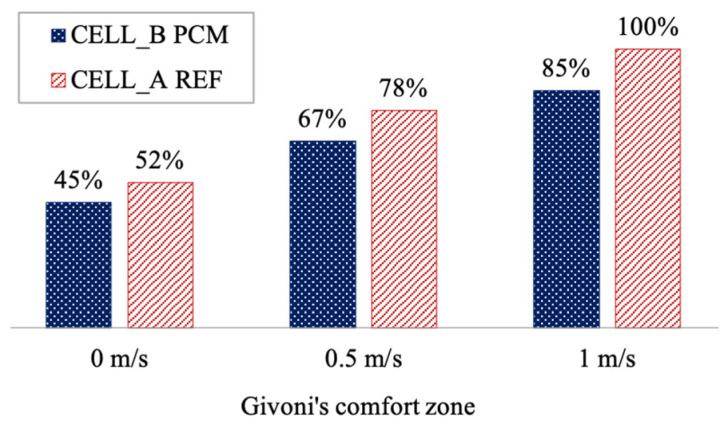
Givoni’s bioclimatic chart for the summer day.

Now, we aim to illustrate the potential impact on the theoretical thermal comfort of an occupant using the Givoni comfort ranges. We plot the operating temperature and relative humidity on a psychrometric diagram alongside the three Givoni summer comfort zones (Figure 16).

This examination reaffirms the findings of our earlier analysis: the PCM appears to noticeably reduce the proportion of time spent in thermal comfort conditions, with a difference of nearly 7% compared to cell A in a non-ventilated room scenario.

In a lightly ventilated room (at 0.5 m s^−1^), the PCM-coated product achieves a state of thermal neutrality for 67% of the time, whereas it reaches 78% without the PCM. When considering ventilation at 1 m s^−1^, the PCM product does not achieve total comfort, unlike the reference cell.

Over a 24 h measurement period, the PCM coating provides thermal comfort for 11 h instead of 12 h without the PCM and without ventilation. The differences become more pronounced with stronger ventilation (1 m s^−1^): the PCM reduces the duration of optimal environmental conditions conducive to thermal neutrality for individuals by 4 h.

Both the segmented analysis and the use of the Givoni bioclimatic chart highlight the unsuitability of this PCM for the summer climatic conditions in Tampon. Cell B with the PCM is warmer than the reference cell, despite its positive influence on relative humidity. Even with optimized ventilation, complete equilibrium is difficult to achieve. Potential avenues for improvement could involve adjusting the intrinsic parameters of the PCM, such as its temperature and phase change enthalpy, as well as increasing the PCM’s surface area and/or thickness.

#### 4.4.4. Winter Day: 23 August 2021

**Figure 17:**
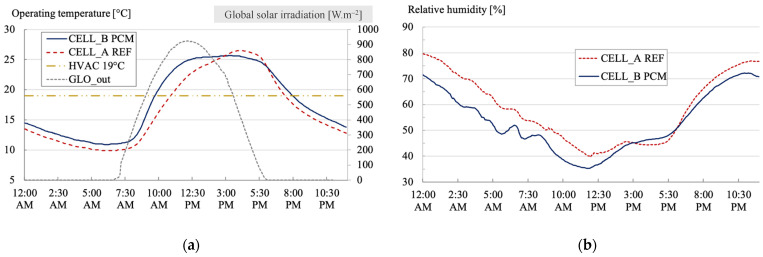
Evolution of operating temperature (**a**) and relative humidity (**b**) inside the test benches during the winter day (Cell A: without the PCM; Cell B: with the PCM).

On 23 August, characterized by clear skies and maximum global solar irradiation, nearly reaching a nominal value of 930 W/m^2^, we conducted a similar analysis as before. We first segmented the data and then examined the thermal comfort conditions within the two cells. The winter season in Le Tampon is known for its significant day/night temperature fluctuations, often exceeding 17 °C. On 23 August, the average outdoor air temperature was 13.7 ± 6 °C. Consequently, the operating temperatures inside the cells were lower than during the summer. The same trend observed during the summer day continues: the operating air temperatures are generally higher in cell B than in the reference cell (Figure 17). They averaged 17.9 °C for cell B, compared to 16.8 °C without the PCM. The maximum temperature recorded in the reference cell was 26.5 °C, while it reached 25.7 °C with the PCM. The maximum daily temperature range is therefore narrower in the cell with the PCM (14.8 °C) than in the reference cell (16.6 °C). This behavior is characteristic of a more thermally insulated cell.

Regarding relative humidity, the PCM coating appears to positively influence the indoor environment by effectively regulating the moisture content in the air (Figure 17). On average, relative humidity measures approximately 52.9% with the PCM compared to 58.1% without. The maximum relative humidity levels reached in the cell with the PCM are 72.3%, as opposed to 79.7% without. On the other hand, the minimum relative humidity recorded in cell A is 35.2% with the PCM, while it is about 39.6% without. In winter conditions, relative humidity increases thermal exchanges between occupants and their surroundings, potentially impacting thermal comfort.

Despite its limited thickness and surface distribution within the room, the PCM’s hygroscopic behavior is again evident. The PCM could offer significant economic benefits if the cells were equipped with an active heating system (with a temperature set at 19 °C; see Figure 17). In this scenario, the PCM would positively affect HVAC power consumption. As temperatures are generally higher in cell B, the heating solution’s operational duration would be reduced by 14 h with the PCM compared to 16 h for the reference cell.

**Figure 18:**
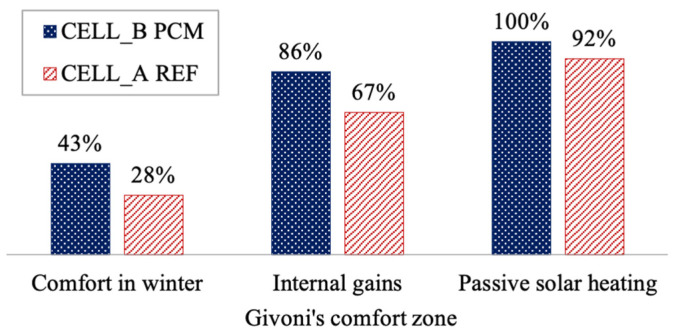
Givoni’s bioclimatic chart for the winter day.

Now, shifting our focus to the thermal experience of a hypothetical occupant, we can demonstrate that the PCM increases the daily duration of thermal neutrality (Figure 18). The Givoni chart for winter conditions suggests that comfort ranges can be extended by incorporating passive heating solutions such as internal load addition or passive solar heating.

The PCM significantly enhances the proportion of time spent in winter thermal comfort and passive conditions, with an increase of nearly 15% compared to the cell without the PCM. In a room with internal loads, the product can achieve a state of thermal neutrality for 86% of the time, as opposed to 67% for cell A. Total thermal comfort is attained when the PCM is combined with passive solar heating solutions, such as a Trombe wall.

Over a 24 h measurement period, the PCM coating enables thermal comfort for 10 h, compared to 7 h without the PCM. The impact is even more significant when internal loads are present: the PCM extends the optimal environmental conditions for thermal neutrality by 5 h.

This product appears to be better suited to winter conditions in Le Tampon than in the summer. To further validate this finding, we aim to analyze more extended periods.

#### 4.4.5. Seasonal Studies on the PCM

In this section, we aim to extend thermal comfort analysis across periods of significant amplitude. This approach allows us to assess the overall potential of the PCM coating without being constrained to a specific day, thereby avoiding a focus on isolated events.

**Figure 19:**
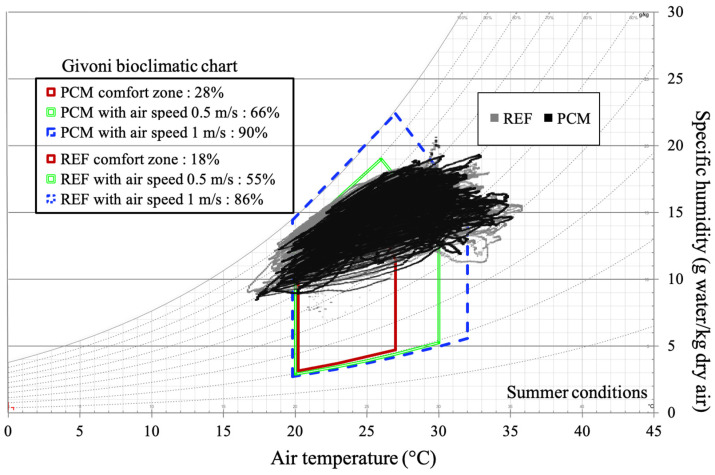
Givoni’s bioclimatic chart for the summer season.

The summer season corresponds to period 1, as described in Figure 14. It is characterized by a relatively cool climate, with an average air temperature of 21.6 °C, fluctuating between 28.8 °C and 15.6 °C. The climate in this region can be described as tropical, verging on temperate. We have integrated all data points for the measurement period with a one-minute time step, encompassing more than 2400 h of data. In contrast to the daily analysis, examining the data over the entire summer period provides a fresh perspective on the performance of the PCM coating (Figure 19). In this case, the advantage clearly favors the PCM, regardless of the comfort zone considered.

**Figure 20:**
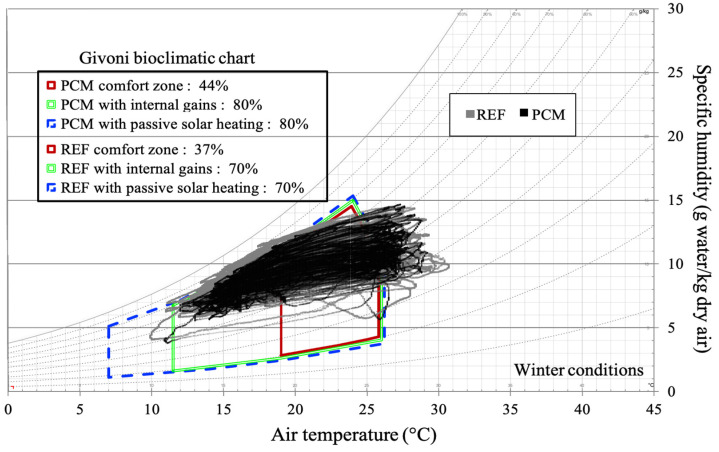
Givoni’s bioclimatic chart for the winter season.

Without ventilation, the PCM enables thermal comfort conditions for 28% of the time, equivalent to more than 672 h of energy autonomy, compared to only 10% for the reference cell. The performance decreases slightly as airspeed increases, with differences of 11% and 4% for ventilation at 0.5 m s^−1^ and 1 m s^−1^, respectively. However, the results do not indicate a significant adaptation to varying ventilation rates. The winter season, corresponding to period 2 in Figure 14, is characterized by average temperatures around 16.8 °C, nearly 5 °C lower than in the summer. Our study covers 2362 h of instrumental observation during this season. The point cloud depicted in Figure 20 reveals a narrower range of humidity levels in the air for the cell with the PCM compared to the reference cell. This notably impacts the thermal comfort rates within the Givoni comfort zones. The use of PCM alone extends the period of thermal neutrality by 7%. When combined with internal loads or solar heating solutions, the effect on thermal comfort occurrence remains consistent, with a constant deviation of 10% compared to cell A. In summary, our decoupled analysis of parameters and the assessment of comfort levels over typical days and extended periods have allowed us to discern the potential of PCM technology based on a cellulosic matrix. The most striking result is the impact on relative humidity, underscoring the need for better adaptation of the intrinsic parameters of the PCM and its integration with a matrix volume that is in harmony with the air volume to be treated.

## 5. Conclusions

The thermal performance of PCMs is often claimed to be closely linked to their chemical structure and composition. Using a commercial PCM, the first step in our research was to determine the composition of this material, both chemically and structurally. Then, the thermal properties of this material were described on a laboratory scale. These preliminary experiments provided a sound scientific basis for the study of the material’s macroscopic thermal performance on various test platforms and in specific climatic environments.

More particularly, our multi-scale analysis highlights the significant potential of Phase Change Materials (PCMs) as an essential element for improving thermal comfort in tropical buildings. The PCM studied demonstrated a consistent ability to reduce daily and seasonal temperature fluctuations, confirming its effectiveness in stabilizing indoor climates.

But, our results emphasize the need for meticulous selection of materials to meet a region’s microclimatic requirements. In other words, no universal compound can satisfy all climatic environments. Moreover, it is vital to identify phase change temperatures suited for diurnal and seasonal thermal profiles.

This research also stresses that the integration of PCMs must be approached holistically, considering factors such as PCM volume relative to air volume and prevailing climatic conditions. This reveals practical implications for modulating heat flux and stabilizing indoor temperatures against seasonal fluctuations. This approach can contribute to reducing the need for active cooling and heating systems commonly found in buildings in tropical regions, without reducing the level of thermal comfort expected. Additionally, this approach can be applied at a broader scale under other climatic conditions.

Our work offers new and highly interesting perspectives for study. Indeed, it would be very interesting to be able to go further on the correlation rules between phase change temperature and comfort periods. On a practical level, it would also be relevant to study the relationship between the volume of the PCM and the volume of air to be taken into account. This work will necessarily be based on inter-climatic validation in order to offer real prospects for applications. It will also guide the development of useful standards based on concrete results and be capable of guiding the housing sector towards sustainable construction practices. Finally, to take the development of environmentally friendly solutions even further, it would be interesting to also work on the scientific and technical qualification of biosourced Phase Change Materials. In fact, such a study has recently been undertaken by our multidisciplinary research team, exploring a wide range of vegetable oils [97]. A future based on greener solutions seems possible.

## Figures and Tables

**Table 1:** Physical properties of the construction materials (according to the supplier’s documentation: “SYSTEM PRT PUR Bs3d0”).

Material	Surface Transmittance (W m^−2^ K^−1^)	Density (kg m^−3^)	Thickness (m)
Composite wall (galvanized steel, polyurethane foam, galvanized steel)	0.58	40	0.4

**Table 2:** Selected days for the study.

Day	Outdoor Air Temperature Range (°C)	Reference Cell Air Temperature Range (°C)	Diffuse/Global Irradiation Ratio
23 August 2021 (winter)	8.4–20	10–27	0.11
15 October 2021 (mid-season)	13.7–27.6	15.5–31.6	0.1
26 February 2021 (summer)	19–26.2	20.4–31.7	0.32

**Table 3:** Exploitation of DSC results.

Heating Rate (°C min^−1^)	Process	Onset (°C)	Peak (°C)	Endset (°C)	Enthalpy (kJ kg^−1^)
5	Melting	26.9	35.7	39.6	160
−5	Solidification	25.3	17.8	12.4	157
3	Melting	26.8	33.6	36.5	162
−3	Solidification	25.7	19.9	16.2	160
1	Melting	26.6	30.2	32.1	167
−1	Solidification	26.0	22.7	20.4	161
0.3	Melting	26.9	29.0	29.9	171
−0.3	Solidification	26.4	24.4	22.8	161

**Table 4:** Endset temperatures evaluated by graphical visualization.

Date	② Melting Endset (°C)	④ Crystallization Endset (°C)
26 February 2021 (summer)	28.3	23.4
15 October 2021 (mid-season)	28.5	22.2

**Table 5:** Comparison of endset temperature evaluation methods.

Date	Melting Endset (°C)			Crystallization Endset (°C)		
	Graphical ②	DSC (*)	Absolute Difference	Graphical ④	DSC (*)	Absolute Difference
26 February 2021 (summer)	28.3	29.9	1.6	23.4	22.8	0.6
15 October 2021 (mid-season)	28.5	29.9	1.4	22.2	22.8	0.6

(*): values taken from Table 3 for a heating/cooling ramp of 0.3 °C/min.

## Data Availability

The original contributions presented in the study are included in the article, further inquiries can be directed to the corresponding authors.
